# Guy’s Hospital—Pneumonia, Bleeding, Rapid Recovery

**Published:** 1869-01

**Authors:** 


### Guy's Hospital—Pneumonia, Bleeding, Rapid Recovery.

[Under the care of Dr. Wilks.]

We quote the following case because of the remarks upon blood-letting which were made by Dr. Wilks, and which will doubtless be read with interest at the present time, when discussion upon the utility or disadvantage of the practice is again being revived:

Louisa B——, aged eighteen, a servant girl, small but robust, was admitted September 23d. Two days before she became rather suddenly ill, and after a few hours was worse, with a pain in the right side. On admission she evidently had pneumonia; the skin was very hot, the face flushed, and there was a catch in the side on breathing. On examination, there was dullness at the lower part, with bronchophony and a slight pleuritic rub; rusty expectoration. Dr. Wilks remarked that the case was not very severe, and he was unable to tell whether the inflammation would advance or would stay at its present site; he therefore merely ordered some saline and Dover's powder. On the following day she was worse, and towards evening was excessively ill; the fever was very high, and there was great oppression of breathing; crepitation was heard all over the remainder of the right lung, and there was some suspicion of the left lung having been also attacked. Mr. Reginald Stocker having informed Dr. Wilks of her condition, the latter ordered her to be bled, and to have calomel, antimony, and opium pill every four hours. This was accordingly done, and with very marked and quick relief to her oppressed breathing. On the following day she was much more comfortable; bronchophony now heard at top of lung; the left quite free. On September 26th the febrile symptoms were subsiding; physical signs of consolidation of lung pretty perfect. On the 28th, much better; fever departing; sputa bronchial; to omit the pills. In a day or two she left her bed, rapidly convalesced and quitted the hospital quite well on October 14th.

Dr. Wilks remarked that his opportunities were small for treating pneumonia at the onset of the attack, and therefore he was generally obliged to do little more than watch a case in its progress; he had not, however, given up the idea of being able to arrest it by remedies. He stated that he had seen a great deal of pneumonia in private practice during the last summer—in fact, more cases than during the whole of the two preceding years; and what had struck him as most remarkable was the circumstance that nearly all were alike. Thus in all but two the right lung was the organ affected, the inflammation commencing below and progressing upwards; and in nearly all there was a total absence of expectoration. In nearly every case stimulants were given. All but three recovered. One very bad case, that of a lady aged sixty, did well without the use of stimulants. Dr. Wilks could not draw any important inference from the treatment adopted. He believed the majority of cases would have done on any method; that one or two were benefited by stimulants, and that more than one case was aggravated by their use. If it be true, and no doubt it is, that the tendency is towards recovery if no medicine be given, the question should be, not whether a case does well with bleeding or with brandy, but whether the abstraction of a pint of blood, or the administration of a few ounces of alcohol daily, is sufficient to kill the patient.

As regards blood-letting, Dr. Wilks said he had no data on which he could found an opinion as to its value in pneumonia or other diseases, as an antiphlogistic—that is, as to its power in arresting the inflammatory processes; but he had no doubt as to its good effects in relieving congestion of the lungs under any circumstances. He believed therefore, firmly, that he had seen venesection save life in pneumonia, bronchitis, heart disease, apoplexy, or epilepsy. In pre-auscultatory times it might be that the doctor was apt t0 style many chest affections pneumonia; but when he was called to a patient sitting up in a chair, livid in the face, panting for breath, and he took out his lancet, and whilst he was bleeding *pleno rivo* he saw tranquility restored, he could not be mistaken as to the good effects of his remedy. Thus it was in the case above reported; immediate relief was obtained, and it may be as Mr. Stocker thought, an arrest to the further progress of the inflammation.

As regards the other old-fashioned remedies for pneumonia, Dr. Wilks stated that he had no doubt opium could arrest inflammation, as the fact could be observed on the outward parts of the body; and he thought there was good reason for believing that antimony had the same tendency. As regards calomel, he considered it a valuable remedy in many morbid conditions, but was not aware of any facts to prove that it had any power of arresting inflammation. Blisters he had long ago given up using in pneumonia. He was not at all inclined to despair of other remedies of the opium class, which might arrest inflammatory action; and as aconite had long had the character of possessing this property, he had been using it whenever an early stage of acute disease came under his notice. Thus he had had a case of pneumonia, and two or three of rheumatic fever, where the symptoms rapidly abated during its use. Why he believed the remedy was efficacious was, that a most remarkable lowering of the pulse took place after the administration of a few doses.—*London Lancet.*

